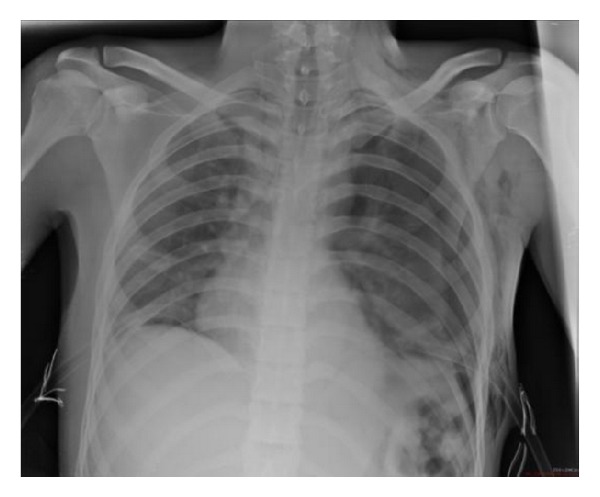# Bilateral Chylhotorax after Falling from Height

**DOI:** 10.1155/2014/618708

**Published:** 2014-06-26

**Authors:** Vildan Akpinar, Fulya Yilmaz Duran, Elif Duman, Murat Yasar Özkalkanli, Özgür Duran, Burcu Horsanali

**Affiliations:** ^1^Anaesthesiology and Reanimation Department, Bozyaka Training and Research Hospital, İzmir, Turkey; ^2^Thoracic Surgery Department, Bozyaka Training and Research Hospital, İzmir, Turkey; ^3^Emergency Medicine Department, Bozyaka Training and Research Hospital, İzmir, Turkey

## Abstract

Chylothorax is accumulation of chyle in the pleural cavity due to disruption of the thoracic duct. The causes can be classified as neoplastic, traumatic (iatrogenic or noniatrogenic), congenital, sporadic, spontaneous, and miscellaneous. A 22-year-old man with no feature in his history and family history was referred to emergency department with the case of falling from height. Abdominal computed tomogram (CT) revealed laceration of liver, grade 5 splenic laceration, fracture of the left acetabulum, and dislocation of the left hip. He was optimized for emergency splenectomy and close left hip reduction. On the 2nd day of the operation, bilateral chylotorax revealed. The treatment depends on its etiology, the amount of drainage, and the clinical picture. Treatment can be classified into 3 categories treatment of the underlying condition, conservative management (such as bed rest, nil by mouth or low fat medium chain triglycerides by mouth and total parenteral nutrition), and surgical management by ligation or clipping of the thoracic duct with open thoracotomy or video-assisted thoracoscopic surgery. The main purpose of surgical treatment is to stop the chylous leak.

## 1. Introduction

Chylothorax is accumulation of chyle in the pleural cavity due to disruption of the thoracic duct [1–4]. Chylothorax is rare following blunt trauma; its diagnosis is usually delayed until the puncture or drainage of posttraumatic pleural effusion and its cause is not clear [5, 6]. The causes can be classified as neoplastic, traumatic (iatrogenic or noniatrogenic), congenital, sporadic, spontaneous, and miscellaneous [1, 3, 4]. We assumed bilateral chylothorax in this case was related to the fall from height.

## 2. Case Report

A 22-year-old man was referred to our hospital after falling from height. He has no feature in his history and family history. He was conscious and cooperative. Vital signs and laboratory tests were within normal limits. Abdominal ultrasonography revealed free fluid in perisplenic and perihepatic areas and Morrison pouch. It is more prominent in the perisplenic area. Abdominal computed tomogram (CT) revealed laceration of liver, grade 5 splenic laceration, fracture of the left acetabulum, and dislocation of the left hip; brain CT revealed air-fluid level in right maxillary sinus and both sphenoid sinuses, upper and medial wall fracture of right orbita; thorax CT revealed parenchymal laceration and contusional changes. He was optimized for emergency splenectomy and close left hip reduction.

**Figure 1:**
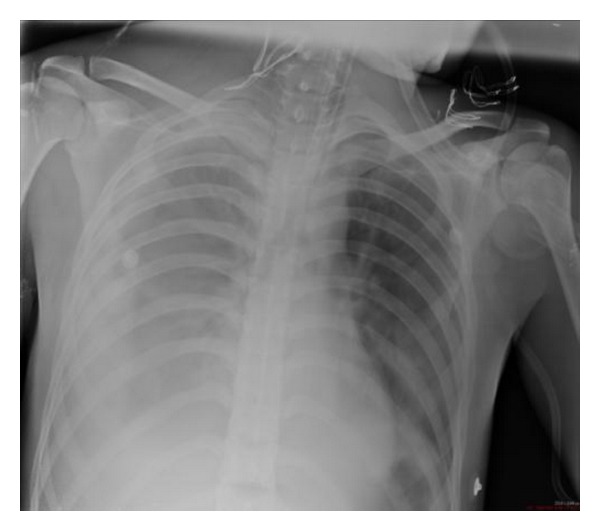


Preoperative laboratory findings were as follows: WBC: 23600/mm^3^, Hb: 12.6 g/dL, Htc: % 44, platelets: 292000/mm^3^, glucose: 141 mg/dL, BUN: 22 mg/dL, Cr: 0.99 mg/dL, Na^+^: 140 mEq/L, K^+^: 2.37 mEq/L, and Cl^−^: 105 mEq/L. Postoperatively the patient was admitted to the intensive care unit. On the 2nd day of the operation, he was extubated without any problem. Right pleural fluid was detected in control chest X-ray after extubation (Figure 1). The patient was examined by thoracic surgeon and chest tube was placed on the right side. The appearance of the fluid was chylous which was later confirmed on biochemical analysis (triglyceride level: 90 mg/dL; cholesterol level: 7 mg/dL). Conservative treatment for chylothorax with nothing by mouth and total parenteral nutrition (TPN) was instituted. On the 3rd day of the operation, thorax CT showed right pleural fluid and chest tube was 2 placed on the right side. The appearance of the fluid was chylous which was later confirmed on biochemical analysis (triglyceride level: 245 mg/dL; cholesterol level: 15 mg/dL). On the 4th day of the operation chest X-ray showed left pleural fluid and chest tube was placed on the left side. on the 10th day of the operation, the patient was discharged to the ward. On the 11th day of the operation, although left basal tube was inserted, left total pneumothorax (Figure 2) was developed and left apical tube thoracostomy was performed. Left apical chest tube was removed 6 days after insertion and left basal chest tube was removed 13 days after insertion. On the 18th day of the operation, although right basal tube was inserted, control chest X-ray showed right total pneumothorax and right apical tube thoracostomy was performed. After right apical tube thoracostomy was performed, right basal chest tube was removed 16 days after insertion. Right apical chest tube removed 6 days after insertion. The patient was discharged from hospital on the 28th day of operation without any complication.

## 3. Discussion

In this paper, a case was reported of a man who fell from height and was operated for splenic laceration and developed bilateral chylothorax on the 2nd day of the operation. Although chylothorax due to blunt trauma was reported in the literature, this patient has developed chylothorax on the left side one day after the detection of right side chylothorax.

T**horacic duct** is the main lymphatic duct that binds lymphatic system to venous system. Its function is to transport lipids absorbed from the gastrointestinal tract. Traumatic noniatrogenic chylothorax etiology ratio is 2.6% [6]. The most common form of blunt injury to the thoracic duct is produced by sudden hyperextension of the spine with rupture of the duct just above the diaphragm in the right thorax. Half of the chylothoraces are right-sided, one-third is left-sided, and the remainders are bilateral [4]. This patient encountered trauma after falling from height. Damage to his thoracic duct most likely occurred as a result of sudden hyperextension of the spine. The colour of the pleural fluid is not always indicative of chylothorax. It may not appear chylous if the patient is fasting or the pleural fluid is mixed with blood [4]. There are several techniques [presence of chylomicrons in the pleural fluid [3, 4, 7], lipoprotein analysis [4], bipedal lymphangiography [4], and analysis of the pleural fluid [3, 4, 7]] used in the diagnosis of chylothorax. The exact diagnosis of chylothorax is based on the presence of chylomicrons in the pleural fluid [3, 4]. Important 3 characteristics of the fluid are pH of 7.4 to 7.8 [4], lymphocyte predominance in cell count [7], specific gravity of 1.012 or higher [4], triglyceride levels > 110 mg/dL [3, 4, 7], and low cholesterol level [7]. Clinical features are hypovolemia, respiratory difficulty, malnutrition, immunosuppression, dyspnea, chest pain, and cough [3]. Symptoms of chylothorax typically have a gradual onset. Often, a latency period of 2–7 days exists between the time of injury and clinical evidence of chylothorax [4]. The longest latency period reported in the literature was 20 years [1]. In our case, the latency period is 2 days. However, this patient had developed chylothorax first on the right side and one day later on the left side. The treatment depends on its etiology, the amount of drainage, and the clinical picture [4]. Treatment can be classified under 3 categories: treatment of the underlying condition, conservative management (such as bed rest, nil by mouth or low fat medium chain triglycerides by mouth, and total parenteral nutrition), and surgical management by ligation or clipping of the thoracic duct with open thoracotomy or video-assisted thoracoscopic surgery [3, 4]. Somatostatin and octreotide have proved to be useful in the conservative treatment of chylothorax [3, 7]. Conservative measurements have achieved an 88% success rate [4]. The duration of conservative management varies in the literature anywhere from 1 to 8 weeks [6], but most authors recommend conservative management for no more than 2 weeks [4].

The main purpose of surgical treatment is to stop the chylous leak [6]. In general, surgical intervention offers better results than conservative management when daily chyle leak exceeds 1 L/day for a period more than 5 days or 1.5 L/day in an adult or > 100 mL/kg body weight per day in a child, persistent chyle flow for more than 2 weeks [3, 4]. Surgery is also recommended if here has been a rapid decline in nutritional status despite conservative management [3].

Before the introduction of surgical ligation of the thoracic duct, the mortality rate from chylothorax was approximately 100% [6]. With the advent of TPN and surgical ligation for persistent leaks, the mortality rate of chylothorax became less than 10% [4].

This case is a rare example of bilateral chylothorax resulting from falling from height and onset of bilateral pneumothorax in a patient with open and functioning bilateral basal chest tubes. The mechanism of chylothorax is thought to be a hyperextension injury to the spine. But we cannot explain the onset of bilateral pneumothorax.

The pleural fluid was chylous and contained high triglycerides concentration and low cholesterol level. We performed a conservative approach including tube thoracostomy, nil by mouth, and TPN, which resulted in regression of the chylous fluid and improvement of the patient. Medium chain triglycerides were not given to patient but TPN was applied to prevent malnutrition due to chylothorax and fasting.

In conclusion, early detection of the bilateral chylothorax due to blunt trauma resulted in early management. During the ICU stay daily imaging demonstrated the delay of left-sided chylothorax one day after the right-sided chylothorax. Conservative management resulted in successful treatment.

## Figures and Tables

**Figure 2:**